# Efficacy of Adjunct Hemoperfusion Compared to Standard Medical Therapy on 28-Day Mortality in Leptospirosis Patients with Renal Failure and Shock: A Single-Center Randomized Controlled Trial

**DOI:** 10.3390/tropicalmed9090206

**Published:** 2024-09-09

**Authors:** Danice Romagne Leano, Romina Danguilan, Mel-Hatra Arakama, Vince Apelin, Paolo Pinkerton Alamillo, Eric Chua

**Affiliations:** Department of Adult Nephrology, National Kidney and Transplant Institute, Quezon City 1101, Philippines

**Keywords:** cytokine storm, hemoperfusion, leptospirosis, mortality, septic shock

## Abstract

Hemoperfusion is a novel adjunct therapy that targets the dysregulated inflammatory events in severe sepsis. Previous studies have reported conflicting results on its efficacy and safety. This study was designed to assess the efficacy and safety of hemoperfusion among leptospirosis patients in septic shock and renal failure in terms of improvement in 28-day mortality, SOFA score, level of inflammatory markers, hemodynamics, and renal and pulmonary function. A total of 37 severe leptospirosis patients were enrolled and randomized into either standard medical therapy (SMT) alone, *n* = 20, or with hemoperfusion (HP), *n* = 17. Vital signs, urine output, vasopressor dose, PaO_2_/FiO_2_ (P/F) ratio, and biochemical parameters of patients from each treatment arm were compared. The hemoperfusion group showed a 36.84% (*p* = 0.017) risk reduction in 28-day mortality. Levels of procalcitonin, IL6, and lactate significantly decreased from baseline to day 7 in both groups. Statistically significant improvements in serum creatinine (*p* = 0.04) and PF ratio (*p* = 0.045) were observed in the hemoperfusion cohort. Intention-to-treat and per-protocol approaches showed that hemoperfusion increased the survival rate and decreased the mortality risk. This benefit for survival persisted even when patients were also receiving extracorporeal membrane oxygenation (ECMO), showing that hemoperfusion’s benefits are independent of ECMO use. Hemoperfusion is a safe and effective adjunct therapy for managing severe sepsis. It promotes earlier renal and pulmonary function recovery and improves the survival of septic shock patients.

## 1. Introduction

Sepsis and septic shock continue to be major contributors to global mortality, with approximately six million deaths occurring annually despite advances in diagnostic methods and the availability of a broad spectrum of antimicrobials [1,2,3]. The Society of Critical Care Medicine’s 2016 definition characterizes sepsis as a life-threatening organ dysfunction resulting from a dysregulated host response to infection, which can lead to septic shock, multi-organ dysfunction, and increased mortality rates [2,3,4]. The mortality rates associated with sepsis and septic shock vary due to factors such as disease severity, geographic location, and standards of care [1,2,3].

Hemoperfusion has gained attention as an adjunctive therapy for managing sepsis and septic shock because of its potential to remove circulating cytokines and other inflammatory mediators. The rationale for hemoperfusion lies in its ability to mitigate the cytokine storm characteristic of severe sepsis, thereby reducing the risk of multi-organ dysfunction and improving patient outcomes. Despite these theoretical benefits, the clinical efficacy of hemoperfusion remains under investigation, with varying results reported across studies. Hemoperfusion is an extracorporeal blood purification technique designed to remove toxins from the bloodstream using a cartridge filled with adsorbent materials that selectively capture and eliminate specific cytokines. The cartridge employs neutral microporous beads with a pore size distribution optimized for removing inflammatory mediators associated with sepsis-induced organ dysfunction [5,6,7]. Although studies on the efficacy of hemoperfusion in improving survival outcomes for septic patients have yielded conflicting results, its potential to enhance outcomes in sepsis warrants further investigation.

Huang and colleagues evaluated septic patients using a neutral microporous resin column, finding a significant reduction in mortality. This study, which involved 100 patients, demonstrated a 38.7% reduction in mortality in the hemoperfusion group compared to controls, suggesting the potential for hemoperfusion to improve survival in septic shock by removing humoral mediators from the bloodstream [8].

Further research by Chu et al. expanded on these findings by combining hemoperfusion with high-volume hemofiltration in a multi-center study of septic shock patients. The study reported a 13.3% reduction in 28-day mortality among patients who received the combined therapy compared to those who received standard care alone. Improvements in hemodynamic stability and reductions in inflammatory markers such as IL-6 were also observed, supporting the role of hemoperfusion in improving the prognosis of septic shock patients [9].

In Japan, Lee et al. focused on using polymyxin B hemoperfusion in septic shock patients. This study, involving 64 patients, reported improvements in hemodynamic parameters and a trend toward reduced mortality, although the difference was not statistically significant. These findings contribute to the growing body of evidence suggesting that hemoperfusion could be beneficial in managing septic shock, particularly in cases involving Gram-negative bacterial infections [10].

Research from Turkey has further explained the role of hemoperfusion in sepsis management. Kaçar et al. assessed the efficacy of the HA330 hemoperfusion cartridge in patients with septic shock and acute kidney injury (AKI). This study, which involved 42 patients treated with continuous venovenous hemodiafiltration (CVVHDF) combined with hemoperfusion, showed a significant reduction in serum cytokine levels and improved survival rates in the hemoperfusion group. The authors concluded that hemoperfusion could serve as an adjunct therapy in critically ill patients with sepsis and AKI. However, they emphasized the need for larger randomized controlled trials to validate these findings [11].

In Italy, Ronco et al. explored the role of extracorporeal blood purification techniques, including hemoperfusion, in managing acute kidney injury (AKI) in septic patients. Their review highlighted the potential of these techniques to modulate the immune response in sepsis, particularly in patients with AKI. However, they also noted that the effectiveness of hemoperfusion varied depending on factors such as patient population, timing of intervention, and the specific device used, demonstrating a need for more standardized approaches to optimize outcomes [5].

A systematic review and meta-analysis by Putzu and colleagues further evaluated the impact of blood purification techniques, including hemoperfusion, on sepsis and septic shock mortality. This analysis included 21 randomized controlled trials encompassing 2073 patients. Although the review found that these techniques could reduce circulating inflammatory mediators, the evidence for a mortality benefit was inconclusive. The authors underscored the need for more rigorous studies to determine the optimal use of these therapies [4].

Despite the promising results reported, outcome variability persists. Factors such as patient selection, timing of therapy, the specific hemoperfusion device used, and the duration of treatment all appear to influence the intervention’s effectiveness. Studies are limited by small sample sizes and single-center designs, underscoring the necessity of larger, multi-center randomized controlled trials to establish definitive evidence.

Leptospirosis, a widespread zoonotic infection in the Philippines, serves as a classic example of sepsis-induced multi-organ dysfunction in its most severe form. Southeast Asia’s estimated morbidity and mortality rates are significantly higher at 55.54 and 2.96 per 100,000 population, respectively [12]. In 2016, Pasamba and colleagues reported that the majority of leptospirosis-related deaths at the National Kidney and Transplant Institute (NKTI) were due to a fulminant pulmonary hemorrhage. Acute renal failure also contributes to leptospirosis-related morbidity, with most cases recovering after renal replacement therapy [13].

The treatment protocol for severe leptospirosis at NKTI incorporates a comprehensive approach focusing on antibiotics and organ-specific supportive measures, which are crucial for managing sepsis and septic shock. The protocol begins with fluid resuscitation and empiric antibiotic therapy using either Penicillin or Ceftriaxone. Vasopressor support is provided as necessary, along with oxygen supplementation based on the patient’s respiratory requirements.

Supportive renal replacement therapy is administered when needed. For patients receiving vasopressors, showing infiltrates on chest X-rays, or having an increased clinical risk of bleeding—demonstrated by conditions such as thrombocytopenia or prolonged PT/PTT—a three-day course of Methylprednisolone (250 mg IV) is prescribed. Following this steroid treatment or after any hemoptysis, a single dose of Cyclophosphamide (1 g IV) is administered. This protocol addresses the specific challenges posed by severe leptospirosis, aiming to stabilize the patient and improve outcomes.

The inclusion of Methylprednisolone and Cyclophosphamide as part of the standard treatment for severe leptospirosis complicated by renal failure and pulmonary hemorrhage is well-supported in the literature. These therapeutic agents are crucial in managing the severe inflammatory response that characterizes this life-threatening condition.

Methylprednisolone has been shown to reduce mortality in patients with severe leptospirosis, particularly in cases leading to complications such as pulmonary hemorrhage and multi-organ dysfunction. Kularatne et al. conducted a study in Sri Lanka highlighting the benefits of early administration of high-dose Methylprednisolone [14]. The study recommended a regimen of 500 mg IV for three days, associated with a marked reduction in mortality among severely ill patients. The anti-inflammatory effects of Methylprednisolone help control the cytokine storm in severe leptospirosis, thereby reducing the risk of fatal complications.

Support for methylprednisolone use also comes from a study conducted in the Philippines by Manipol-Larano et al. [15]. This study focused on patients with leptospirosis complicated by renal failure and pulmonary hemorrhage. The results indicate that patients who received a three-day pulse of Methylprednisolone and Cyclophosphamide in addition to standard antibiotics had higher survival rates. This dual approach was particularly effective in controlling the severe inflammatory response and preventing the progression of multi-organ dysfunction.

Cyclophosphamide, an immunosuppressive agent, has also been shown to benefit the treatment of severe leptospirosis, particularly in patients suffering from pulmonary hemorrhage. Trivedi et al. (2009) in India studied the effects of intravenous Cyclophosphamide in treating patients with leptospiral pulmonary alveolar hemorrhage [16]. The study found that Cyclophosphamide suppressed the overactive immune response, which is responsible for severe damage to the pulmonary vasculature in these patients. This suppression helped to reduce mortality and improve patient outcomes.

Manipol-Larano et al. noted the synergistic effect of combining Cyclophosphamide and Methylprednisolone [15]. The study observed that this combination therapy was associated with better control of the immune response and prevention of further organ damage, especially in patients with severe leptospirosis. This treatment strategy, addressing the underlying pathophysiology of the disease, has been shown to enhance recovery and improve survival rates.

In 2021, So and colleagues developed a scoring index termed The-RADS score (Thrombocytopenia, Hemoptysis, RRT, Anuria, Diabetes, and Shortness of Breath) to predict the likelihood of developing pulmonary complications among severe leptospirosis patients, leading to more aggressive management [17]. Since implementing the standard medical therapy (SMT) protocol, NKTI has observed a decline in mortality rates since 2018 despite an overall increase in the number of leptospirosis patients, particularly those requiring dialysis.

This study aims to compare the effectiveness of hemoperfusion using the HA330 cartridge (Jafron Biomedical, Zhuhai, China) with standard medical therapy to improve the 28-day survival rates of patients with septic shock.

## 2. Methodology

This study enrolled 37 adult patients with septic shock. Informed consent was obtained from all participants, randomly assigned to either the standard medical therapy (SMT) alone or the SMT + Hemoperfusion (HP) using the HA330 cartridge (Jafron Biomedical, China) group using permuted block randomization with equal allocation per group. Exclusion criteria included chronic kidney disease, unstable comorbidities before acute illness, non-septic shock, pregnancy, post-cardiac arrest, GCS < 8, or other documented illnesses affecting treatment.

This study used the HA330 hemoperfusion cartridge from Jafron Biomedical because it has proven to be effective in clinical settings and is widely available. The HA330 cartridge uses special beads to remove inflammatory substances from the blood. The mediators played a critical part in the pathophysiology of sepsis-induced multi-organ dysfunction, a severe and common complication in cases of leptospirosis. The HA330 cartridge was chosen for its technical suitability and widespread availability, supported by extensive clinical use and experiential data. Previous research revealed that the HA330 cartridge lowered cytokine levels, such as IL-6, associated with poor outcomes in septic patients.

The researchers recorded baseline scores for APACHE and SOFA scores, along with several laboratory tests. All patients underwent daily intermittent hemodialysis for a minimum of three days. The HP group underwent three hemoperfusion sessions for two hours daily with the HA330 resin cartridge (Jafron Biomedical).

The researchers employed statistical tests, including the Independent Sample *t*-test, Mann–Whitney U test, Fisher’s Exact/Chi-square test, and the Friedman test. Using Stata 15.0 (StataCorp LLC, College Station, TX, USA), the researchers performed intention-to-treat and per-protocol analyses, setting the significance level at α = 0.05.

## 3. Results

The mean age of all participants was approximately 32.65 years, and there was no statistically significant difference observed between the two groups (*p* = 0.381). Gender distribution, body mass index (BMI), and comorbidities such as Diabetes Mellitus, Hypertension, Cardiovascular Disease, Cancer, Pulmonary Tuberculosis, and other ailments were generally absent in both groups. The mean Acute Physiology and Chronic Health Evaluation (APACHE) score, which assesses disease severity, did not significantly differ between the groups (*p* = 0.1085). Additionally, the Sequential Organ Failure Assessment (SOFA) scores, presented as a median with a range, were similar in both groups, with a median SOFA score of 12 for all participants, indicating no significant difference (*p* = 0.613). Oxygen support requirements before admission were also comparable between the groups, with a majority of patients in both not requiring oxygen support upon admission (over 50%). These findings (Table 1) suggest that the groups had a matched baseline characteristics.

This study evaluated the use of HP in addition to SMT compared to SMT alone in 37 patients. Using an intention-to-treat approach, the researchers analyzed the data on day 28 (Table 2). The SMT group had mortality, while the HP group had none. A significant finding from the study was a 36.84% reduction in mortality risk among the HP group (*p* = 0.007), suggesting the potential benefits of HP treatment.

The study also conducted a per protocol analysis, which excluded patients receiving Extracorporeal Membrane Oxygenation (ECMO), and it revealed that none of the HP group died in this subgroup, while the control group’s mortality rate was 36.84% (*p* = 0.017). These results indicated that the improved survival rate in HP group patients was independent of the ECMO use. The researchers terminated the trial prematurely after enrolling more than 50% of the intended sample size, as they saw significant outcomes demonstrating the life-saving potential of HP. The decision was made ethically and was approved by the Research Ethics Committee of the National Kidney and Transplant Institute (NKTI).

Serial monitoring of inflammatory markers and SOFA score of patients showed significant improvement in sepsis score (*p* = 0.018 HP, 0.002 SMT) and levels of procalcitonin (*p* = 0.013 HP, 0.003 SMT), IL6 (*p* = 0.033 HP, 0.020 SMT), and lactate (*p* < 0.001 HP, 0.021 SMT) in both treatment arms from baseline to Day 7.

The vasopressor requirements of patients did not differ significantly whether or not they received hemoperfusion. However, there is clinically greater and earlier reduction in vasopressor score in patients who received hemoperfusion compared with those who received SMT alone Table 3.

Pro-inflammatory cytokines and other inflammatory markers played a crucial role in the pathophysiology of sepsis, where the host’s immune response became dysregulated, leading to widespread inflammation and organ dysfunction. In this study, markers such as IL-6, procalcitonin, and lactate were used to measure inflammation levels in patients and assess the effectiveness of hemoperfusion as a treatment. The levels of procalcitonin, IL-6, and lactate were measured at three-time points: at the start (D0), on day 3 (D3), and day 7 (D7) of the treatment. The results show reductions in these markers from baseline to day 7 in hemoperfusion (HP) and standard medical therapy (SMT) groups. The levels of procalcitonin and IL-6 decreased, suggesting a reduction in inflammation, while lactate levels, which are associated with tissue hypoxia and sepsis severity, also showed improvement (Table 4).

Number of hemodialysis days, urine output and serum creatinine did not significantly differ between the two treatment arms in the intention-to-treat analysis. Based on the per protocol analysis, however, patients who received hemoperfusion showed a statistically significant greater decline in serum creatinine compared with the SMT cohort (Table 5). The latter analysis excluded those who required ECMO who may have sustained greater hypoxic renal injury.

Changes in the P/F ratio did not differ significantly between two treatment arms in the intention-to-treat analysis. However, a statistically significant improvement in the P/F ratio was observed among patients in the HP cohort after excluding patients who received ECMO (Table 5).

## 4. Discussion

Extracorporeal blood purification techniques like hemoperfusion partially clear the body of rogue cytokines responsible for the multi-organ dysfunction and fatal complica-tions of severe sepsis and septic shock.

To date, this is the first local randomized controlled trial to test the efficacy of HA330 hemoperfusion in improving the 28-day mortality of presumptive leptospirosis patients in septic shock and acute renal failure. The mortality risk reduction reported in our study is comparable with the achieved risk reduction in prior studies conducted abroad: 13.3% in the 2019 study by Chu et al. [18] and 38.7% in the 2012 study by Huang et al. [12]. Both studies mentioned above reported a significant decline in IL6 levels in patients who re-ceived HP versus those who did not [12,18].

Our study showed a significant decline in the inflammatory marker level from base-line to Day 7 among patients given hemoperfusion; however, no statistically significant difference was seen compared to the SMT arm. The relatively small sample size of the trial might explain the lack of observed significant change in IL6 levels. Another factor would be the timing of extraction. Levels of inflammatory markers were measured after Day 3 and not immediately after the hemoperfusion session (unlike in studies conducted abroad). It has to be noted that the production of inflammatory cytokines is an ongoing process in sepsis and septic shock. ‘Delayed’ blood extraction translates to higher-than-expected post-hemoperfusion IL6 levels, as the cytokines accumulate during the time off-treatment. Another important consideration is the unavailability of subsequent IL6 levels among the expired patients, all of whom belong to the SMT cohort.

Clinically, an earlier reduction in vasopressor dose is seen in the HP cohort. Howev-er, the beneficial effect of HP on hemodynamics did not achieve statistical significance. The HP cohort showed a statistically significant improvement in both PF ratio and serum creatinine. The HA 330 hemoperfusion benefits the recovery of injured alveolar–capillary barrier permeability and damaged diffusion pathways of oxygen and oxygenation in ex-trapulmonary sepsis-induced lung injury [18]. An earlier recovery of pulmonary function alleviates hypoxic injury to organ systems, including the kidneys, supporting earlier renal recovery. There is no statistically significant difference in the number of dialysis sessions observed between the two treatment arms. Both groups are weaned off of dialysis after an average of three to four sessions. However, it has to be noted that patients who expired (all of whom belong to the SMT cohort) still depended on dialysis on the day of demise. Therefore, more patients in the HP cohort achieved dialysis independence.

Timing is everything. Hemoperfusion removes both pro- and anti-inflammatory cyto-kines. It could either harm the resolution of excess inflammation (removal of an-ti-inflammatory cytokines as they predominate) or cause excessive immunosuppression by attenuating the pro-inflammatory response [19]. If applied too early or too late, hemoperfusion will be counterproductive. Therefore, the level of inflammatory markers is a helpful guide in deciding the timing of hemoperfusion initiation. However, because of its dynamic nature, the level of inflammatory markers alone should not be the sole criterion. Patients presenting elevated inflammatory marker levels and a clinical picture suggestive of them beginning end-organ injury (i.e., infiltrates on chest radiograph or oliguria) will most likely benefit from this adjunct intervention.

Only 72.97% of enrolled presumptive leptospirosis patients were positive for either the Latex Agglutination Test (LAT) or Microscopic Agglutination Test (MAT). Although both have been used as reference assays, they have relatively low sensitivity, as low as 41% in the acute phase of the disease. It is, however, still possible that some of the LAT/MAT-negative patients may have another etiologic agent causing septic shock. Still, the mechanism of action and overall effect of hemoperfusion is not specific for leptospiro-sis patients. Since hemoperfusion acts on the dysregulated immune response characteris-tic of all sepsis-induced multi-organ dysfunction, its applicability to other infectious causes remains.

The main limitation of the study is the small sample size. This study has been pre-terminated for ethical reasons after achieving a favorable outcome using hemoperfu-sion among the study participants. However, since the sample size has been reduced, sta-tistically significant differences in the secondary parameters of interest (i.e., levels of in-flammatory markers vasopressor dose) were not achieved. Involving multiple centers, tar-geting a bigger sample size, and performing death-censored analysis are recommended for future hemoperfusion studies.

## 5. Conclusions

In conclusion, this study found promising results for using hemoperfusion as an additional treatment for patients with septic shock. The data revealed a significant improvement in the 28-day survival rate for patients undergoing hemoperfusion, with a significant reduction in mortality risk. The observed survival benefit was evident in the intention-to-treat analysis and persisted even when accounting for patients concurrently undergoing extracorporeal membrane oxygenation (ECMO), implying that the positive impact of hemoperfusion was not dependent on simultaneous ECMO use.

The HP may be a good addition to other treatments for patients with septic shock because it may help patients survive longer. It is recommended that researchers conduct further studies, including larger-scale trials to validate these findings.

## Figures and Tables

**Table 1:** Demographic profile of patients (*n* = 37).

	Total (*n* = 37)	Hemoperfusion (*n* = 17)	Control (*n* = 20)	*p*-Value
	Mean ± SD; Median (Range); Frequency (%)			
Age, years	32.65 ± 10.77	30.94 ± 8.41	34.20 ± 12.46	0.381 *
Sex				>0.999 ^†^
Male	34 (91.89)	16 (94.12)	18 (90)	
Female	3 (8.11)	1 (5.88)	2 (10)	
BMI	22.98 ± 3.66	23.48 ± 4.74	22.55 ± 2.45	0.451 *
Comorbidities				
Diabetes Mellitus	0	0	0	-
Hypertension	0	0	0	-
Cardiovascular disease	0	0	0	-
Obesity	2 (5.41)	2 (11.76)	0 (0)	0.204 ^†^
Cancer	0	0	0	-
Pulmonary tuberculosis	0	0	0	-
Other comorbidities	0	0	0	-
APACHE score	14.2 ± 2.15	13.5 ± 1.17	15 ± 1.35	0.1085
SOFA score	12 (8–14); [*n* = 37]	12 (9–14); [*n* = 17]	12 (8–14); [*n* = 20]	0.613 ^‡^
O_2_ support before admission (O_2_ support at emergency room)				0.521 ^†^
None (room air)	21 (56.76)	11 (64.71)	10 (50)	
Face mask	11 (29.73)	5 (29.41)	6 (30)	
BIPAP	0	0	0	
Mechanical ventilation	5 (13.51)	1 (5.88)	4 (20)	

BIPAP, Bi-level positive airway pressure. Statistical tests used: *—Independent sample *t*-test; †—Chi-square/Fisher’s Exact test; ‡—Mann–Whitney U test.

**Table 2:** Comparison of 28-day mortality.

	Total	Hemoperfusion	Control	Risk Difference (95% CI)	*p*-Value
	Frequency (%);				
Intention-to-treat	(*n* = 37)	(*n* = 17)	(*n* = 20)		
28-day Mortality	7 (18.92)	0 (0)	7 (35)	−0.35 (−0.56 to −0.14)	0.007
Per protocol	(*n* = 31)	(*n* = 12)	(*n* = 19)		
28-day Mortality	7 (22.58)	0	7 (36.84)	−0.368 (−0.59 to −0.15)	0.017

Statistical test used: Fisher’s Exact/Chi-square test.

**Table 3:** Comparison of vasopressor requirements.

	Total	Hemoperfusion	Control	Mean Difference (95% CI)	*p*-Value
	Mean ± SD				
Intention-to-treat	(*n* = 37)	(*n* = 17)	(*n* = 20)		
Norepinephrine dose Δ_D3–D0_	−0.14 ± 0.63; [*n* = 33]	−0.25 ± 0.34; [*n* = 17]	−0.03 ± 0.83; [*n* = 16]	−0.219 (−0.67 to 0.23)	0.326 *
Norepinephrine dose Δ_D7–D0_	−0.43 ± 0.28; [*n* = 31]	−0.4 ± 0.25; [*n* = 17]	−0.46 ± 0.32; [*n* = 14]	0.054 (−0.16 to 0.26)	0.601 *
Vasoactive score Δ_D3–D0_	−14.39 ± 62.9; [*n* = 33]	−25 ± 34.23; [*n* = 17]	−3.13 ± 83.22; [*n* = 16]	−21.875 (−66.56 to 22.81)	0.326 *
Vasoactive score Δ_D7–D0_	−43.5 ± 28.35; [*n* = 30]	−42.19 ± 24.9; [*n* = 16]	−45 ± 32.76; [*n* = 14]	2.813 (−18.79 to 24.41)	0.792 *
Per protocol	(*n* = 31)	(*n* = 12)	(*n* = 19)		
Norepinephrine dose Δ_D3–D0_	−0.16 ± 0.63; [*n* = 27]	−0.23 ± 0.31; [*n* = 12]	−0.1 ± 0.81; [*n* = 15]	−0.125 (−0.64 to 0.39)	0.620 *
Norepinephrine dose Δ_D7–D0_	−0.42 ± 0.28; [*n* = 25]	−0.35 ± 0.22; [*n* = 12]	−0.48 ± 0.31; [*n* = 13]	0.13 (−0.1 to 0.36)	0.246 *
Vasoactive score Δ_D3–D0s_	−15.56 ± 63.3; [*n* = 27]	−22.5 ± 31.01; [*n* = 12]	−10 ± 81.31; [*n* = 15]	−12.5 (−63.73 to 38.73)	0.620 *
Vasoactive score Δ_D7–D0_	−43.54 ± 27.33; [*n* = 24]	−37.73 ± 21.72; [*n* = 11]	−48.46 ± 31.32; [*n* = 13]	10.734 (−12.52 to 33.99)	0.349 *

Changes were calculated as the value on day 3 or day 7 minus the value on baseline. Statistical test used: *—Independent sample *t*-test.

**Table 4:** Comparison of level of inflammatory markers.

	Total	Hemoperfusion	Control	Mean or Median Difference (95% CI)	*p*-Value
	Median (Range)				
Intention-to-treat	(*n* = 37)	(*n* = 17)	(*n* = 20)		
hsCRP Δ_D3–D0_	−139.53 ± 91.37; [*n* = 32]	−138.52 ± 97.16; [*n* = 17]	−140.67 ± 87.73; [*n* = 15]	2.144 (−65.05 to 69.34)	0.949 *
hsCRP Δ_D7–D0_	−167.28 ± 106.89; [*n* = 30]	−167.21 ± 108.85; [*n* = 17]	−167.37 ± 108.68; [*n* = 13]	0.164 (−81.93 to 82.26)	0.997 *
Procalcitonin Δ_D3–D0_	−12.53 (−324–26.71); [*n* = 32]	−7.35 (−85.72–26.71); [*n* = 17]	−14.97 (−324–−0.08); [*n* = 15]	7.62 (−17.4–32.64)	0.539 ^‡^
Procalcitonin Δ_D7–D0_	−19.24 (−249–−0.46); [*n* = 30]	−10.12 (−193.22–−1.03); [*n* = 17]	−22.54 (−249–−0.46); [*n* = 13]	12.42 (−20.88–45.72)	0.451 ^‡^
IL6 Δ_D3–D0_	−4 (−3956–104); [*n* = 28]	−9 (−3956–22); [*n* = 15]	−3 (−378–104); [*n* = 13]	−6 (−133.74–121.74)	0.924 ^‡^
IL6 Δ_D7–D0_	−14 (−3981–141); [*n* = 24]	−20 (−3981–5); [*n* = 13]	−8 (−380–141); [*n* = 11]	−12 (−149.2–125.2)	0.858 ^‡^
Lactate Δ_D3–D0_	−2.88 (−98–21.5); [*n* = 32]	−2.6 (−28.6–21.5); [*n* = 17]	−4.01 (−98–4.7); [*n* = 15]	1.41 (−5.24–8.06)	0.668 ^‡^
Lactate Δ_D7–D0_	−4.8 (−41–5.6); [*n* = 29]	−4.04 (−33.6–4.8); [*n* = 17]	−8.55 (−41–5.6); [*n* = 12]	6.06 (−0.06–12.18)	0.052 ^‡^
Per protocol	(*n* = 31)	(*n* = 12)	(*n* = 19)		
hsCRP Δ_D3–D0_	−134.89 ± 91.41; [*n* = 26]	−135.6 ± 99.84; [*n* = 12]	−134.29 ± 87.36; [*n* = 14]	−1.313 (−77.06 to 74.43)	0.972 *
hsCRP Δ_D7–D0_	−162.03 ± 102.86; [*n* = 24]	−168.67 ± 105.74; [*n* = 12]	−155.38 ± 104.14; [*n* = 12]	−13.293 (−102.14 to 75.56)	0.759 *
Procalcitonin Δ_D3–D0_	−13.82 (−324–19.77); [*n* = 26]	−13.3 (−85.72–19.77); [*n* = 12]	−13.82 (−324–−0.08); [*n* = 14]	−2.93 (−31.79–25.93)	0.836 ^‡^
Procalcitonin Δ_D7–D0_	−22.04 (−249–−0.46); [*n* = 24]	−16.49 (−193.22–−7.16); [*n* = 12]	−22.04 (−249–−0.46); [*n* = 12]	−0.09 (−48.88–48.7)	0.997 ^‡^
IL6 Δ_D3–D0_	−2.55 (−378–104); [*n* = 22]	−4.5 (−357–22); [*n* = 10]	−2.55 (−378–104); [*n* = 12]	−5 (−128.59–118.59)	0.934 ^‡^
IL6 Δ_D7–D0_	−8 (−380–141); [*n* = 20]	−6.22 (−362–5); [*n* = 9]	−8 (−380–141); [*n* = 11]	1.78 (−135.29–138.85)	0.979 ^‡^
Lactate Δ_D3–D0_	−2.7 (−98–21.5); [*n* = 26]	−1.8 (−28.6–21.5); [*n* = 12]	−5.26 (−98–4.7); [*n* = 14]	4.2 (−3.83–12.23)	0.291 ^‡^
Lactate Δ_D7–D0_	−6.3 (−41–5.6); [*n* = 23]	−5.55 (−33.6–4.8); [*n* = 12]	−7 (−41–5.6); [*n* = 11]	0.7 (−10.78–12.18)	0.900 ^‡^

Changes were calculated as the value on day 3 or day 7 minus the value on baseline. Statistical test used: *—Independent sample *t*-test; ‡—Mann–Whitney U test

**Table 5:** Comparison of results.

	Total	Hemoperfusion	Control	Risk or Mean or Median Difference (95% CI)	*p*-Value
	Frequency (%); Median (Range)				
Intention-to-treat	(*n* = 37)	(*n* = 18)	(*n* = 19)		
Serum creatinine Δ_D7–D0_	−6.17 ± 2.49; [*n* = 30]	−6.77 ± 2.55; [*n* = 17]	−5.39 ± 2.28; [*n* = 13]	−1.38 (−3.22 to 0.46)	0.135 *
PF ratio Δ_D3–D0_	−125 (-429–250); [*n* = 33]	−125 (-429–250); [*n* = 17]	−112 (−428–19); [*n* = 16]	14 (−126.9–154.9)	0.841 ^‡^
Per protocol	(*n* = 31)	(*n* = 12)	(*n* = 19)		
Serum creatinine Δ_D7–D0_	−6.12 ± 2.38; [*n* = 24]	−7.11 ± 2.23; [*n* = 12]	−5.14 ± 2.19; [*n* = 12]	−1.972 (−3.84 to −0.1)	0.040 *
PF ratio Δ_D3–D0_	−85 (−428–250); [*n* = 27]	7.5 (−323–250); [*n* = 12]	−139 (−428–19); [*n* = 15]	144 (3.54–284.46)	0.045 ^‡^

Changes were calculated as the value on day 3 or day 7 minus the value on baseline. Statistical test used: *—Independent sample *t*-test; ‡—Mann–Whitney U test.

## Data Availability

The data supporting the findings of this study are available upon reasonable request. Due to privacy and ethical restrictions, the data cannot be made publicly available.
